# Avian predators taste reject mimetic prey in relation to their signal reliability

**DOI:** 10.1038/s41598-022-05600-5

**Published:** 2022-02-11

**Authors:** R. He, E. Pagani-Núñez, E. Goodale, C. R. A. Barnett

**Affiliations:** 1 Guangxi Key Laboratory of Forest Ecology and Conservation, College of Forestry, Guangxi University, Nanning, Guangxi People’s Republic of China; 2 Department of Health and Environmental Sciences, Xi’an Jiaotong-Liverpool University, Suzhou, Jiangsu People’s Republic of China; 3 Department of Zoology, Graduate School of Science, University of Kyoto, Kyoto, Japan

**Keywords:** Evolution, Zoology, Animal behaviour, Ecology, Behavioural ecology, Evolutionary ecology

## Abstract

Aposematic organisms defend themselves through various means to increase their unprofitability to predators which they advertise with conspicuous warning signals. Predators learn to avoid aposematic prey through associative learning that leads to lower predation. However, when these visual signals become unreliable (e.g., through automimicry or Batesian mimicry), predators may switch from using visual signals to taste sampling prey to choose among them. In this experiment, we tested this possibility in a field experiment where we released a total of 4800 mealworm prey in two clusters consisting of either: (i) undefended prey (injected with water) and (ii) model-mimics (injected with either quinine sulphate [models] or water [mimics]). Prey were deployed at 12 sites, with the mimic frequency of the model-mimics ranging between 0 and 1 (at 0.2 intervals). We found that taste rejection peaked at moderate mimic frequencies (0.4 and 0.6), supporting the idea that taste sampling and rejection of prey is related to signal reliability and predator uncertainty. This is the first time that taste-rejection has been shown to be related to the reliability of prey signals in a mimetic prey system.

## Introduction

When organisms are foraging for food, there is normally variation among food items in their properties such as size, energy content, and level of defence. This variation among food items may increase uncertainty in the feeding organism that could influence its food choices^1^. Other things being equal, predators like to know what they are eating^2,3^. Some organisms defend themselves, often with repellent or toxic chemicals, which they may advertise with conspicuous warning signals^4^. Batesian mimicry and automimicry are types of deception where parasitic mimics copy the warning signals of their defended models (henceforth ‘deceptive mimicry’), which reduces the rate of predation for mimics whilst increasing predation of models. Deceptive mimicry may increase the level of interaction that predators have with prey, thus increasing the predator’s risk of poisoning themselves when attacking potentially defended prey. Predators therefore need to know whether a potentially defended prey item contains toxins^5,6^. Predators may, thus, taste sample mimetic prey to assess whether they are defended when the visual signals of the prey become unreliable^7–9^. Therefore, understanding how predators seek further information and manage uncertainty is a key consideration in understanding predation of mimetic prey systems.

**Figure 1:**
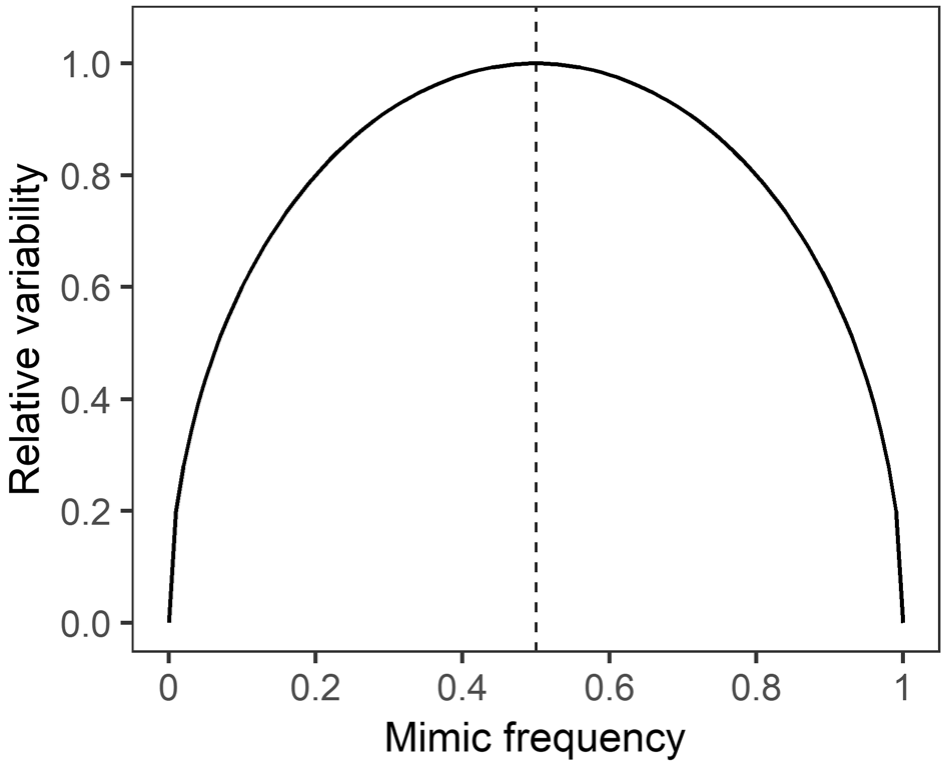
The relative variability in prey defence (y-axis) as a function of mimic frequency (x-axis).

Deceptive mimicry can lead to increased predator uncertainty towards prey^4^. Increases in mimic frequency also increase predation of the models within the prey system^10–16^. Prior to attacking a potentially defended prey, the predator does not know whether it is a distasteful model or an undefended mimic by the visual signal alone. In such situations, predators should proceed with caution and taste sample the prey before deciding to eat or reject the prey (‘go-slow’ behaviour)^17^. Guilford predicted that taste sampling should become prevalent in situations where predators are attacking mimetic prey with unreliable colour signals^17^. We can extend this idea by predicting that the amount of taste rejection should correspond with the level of signal reliability in relation to prey defences in a mimetic prey system (Fig. 1). In a prey system with both models and mimics, the binomial variance has a maximal value at 0.5 and declines at either side of this value. This variance makes it problematic for a predator to predict prey defence of individual prey in cases of deceptive mimicry. Therefore, when mimic frequencies are closer to 0.5, predators may become more uncertain of an individual prey’s defences and therefore taste-sample and reject more prey compared with when mimic frequencies are closer to 0 and 1 (Fig. 1). When mimic frequencies are close to 0, the birds learn that prey are not acceptable and to avoid them. When mimic frequencies are close to 1, the birds learn to generalize that prey are acceptable and to consume them, in both cases without tasting being necessary. Meanwhile, the proportion of attacked prey that were then rejected ought to be highest when there are more models, as has been shown in previous studies [e.g.^18^], because birds that taste sample will reject the bad-tasting models and consume the mimics (see Fig. 1).

In this experiment, we tested the hypothesis that free-living birds would taste-reject more prey when the prey’s colour signals were less reliable at predicting their chemical defences. If our hypothesis reflects a general cognitive response of birds to uncertainty of prey defences, it should be supported across multiple habitats and in multiple species. Therefore, we replicated this experiment in subtropical Nanning, China, and temperate Kyoto, Japan. We predicted that taste-rejection would be highest when mimic frequencies were closest to 0.5, compared with when they were nearer to 0 or 1, and this result would be consistent across sites. However, winter temperatures in Kyoto are colder than in Nanning meaning that birds might be more energetically stressed in Kyoto and more likely to accept distasteful foods than they would if less energetically stressed. This could therefore reduce the level of taste rejection in colder environments^19^. This is the first time a study has examined this prediction across a full spectrum of different mimic frequencies in multiple environments.

## Material and methods

### Study sites

To demonstrate the general applicability of a hypothesis, it is desirable to collect data from sites that differ in their physical characteristics. Climate is one factor that could affect how birds respond to this experiment^19^. Therefore, we conducted this experiment in twelve public parks in two climatic regions, six in subtropical Nanning (Shishan Park [SP], Medicinal Botanical Garden [MBG], Peoples’ Park [PP], Xiangsi Lake Park [XLP], Xinxu River Park [XRP], and Flower Park [FP]) and another six in temperate Kyoto (Yoshidayama [YM], Fudo Onsen [FO], Manshu-in [MI], two areas of Takara-ga-ike [TGI1 and TGI2], and Shimogamo Jinga [SJ]) (see Table S1). Climatic regions were assigned according to the Köppen climate classification^20^. Most sites were more than 2 km from one another within each region to ensure that the bird communities at each site were independent from one another. We chose 10 sub-sites in each park within an area of ~ 4000 m^2^. We used small areas with tree-dominated vegetation, high bird activity, and low human activity as sub-sites. Sub-sites were chosen to have trees or bushes with vegetation within 3 m of the ground to present the prey, with each sub-site being approximately 9 m^2^ in size, and at least 15 m from other sub-sites.

### Prey preparation

We used 20 mm long mealworms (*Tenebrio molitor*) as prey. We treated these prey in different ways which resulted in three different prey types. We had non-treated undefended mealworms that we used during predator training. During the experiment, we used undefended mealworms injected with 0.02 ml of water and defended mealworms injected with 0.02 ml of a 3% quinine sulphate solution (Sigma Aldrich, Q0132–25G).

### Experimental procedure

The experiment had a total duration of 8 days and was comprised of two stages of 4 days each. On the first 4 days we deployed 20 undefended prey at each sub-site (200 prey/site/day), which was a training period that allowed birds to start interacting with the prey. Prey deployed during the training stage were always tied onto 25 × 25 mm blue polypropylene (PP) squares (HTML colour code #81BEF7; https://html-color-codes.info/). The PP colour background acted as an aid to enable the birds to learn the properties of the prey through associative learning^21^. Four days were enough for the birds to find the prey and learn their locations (see Results section).

The second stage (days 5–8) was the experiment itself. During this phase, we deployed prey at the same sub-sites to those used in the training. We deployed prey in two clusters with each cluster being released within an area of 9 m^2^ and at over 1 m from the other cluster. In one cluster, we deployed 10 undefended mealworms (100 controls/site/day) injected with water, which were undefended control prey. We deployed a control to show that predators were able to discriminate among prey (i.e., were educated) and their predation of model-mimics was related to their preferences. In the second cluster, we deployed 10 model-mimics (100 model-mimics/site/day) (the experimental treatment), which were a combination of undefended (injected with water) and defended (injected with quinine) mealworms (for more details of prey preparation and deployment in the field see^21,22^). We used 25 × 25 mm PP squares of different colours for the controls and treatments. We used pink (#F5A9D0) and yellow (#F2F5A9), which were randomly assigned to the control and model-mimic treatments across the 12 sites in a balanced manner (See Fig S1 for what prey looked like, Table S1 for their presentation in the different sites).

Generally, prey within clusters were at least 1 m from other prey within the cluster and clusters were normally situated on adjacent areas on the same tree or on neighbouring trees. The reason the sub-sites were the same between days was for two main reasons. First, the predators needed to learn about the prey and the reliability of their colour signals. This is because the birds would need to become educated about the sites and prey properties. If the location of the prey changed every day, uneducated birds would have been more likely to attack the prey which would reduce the relationship between mimic frequency and taste sampling. Initially, birds would have been less educated about prey and learned about the variability in prey defences from their sampling behaviour. Therefore, clustering of prey and use of the same areas would have aided birds in learning where the prey were if they wanted to interact with them. Second, it logistically made the experiment simpler and it was easier to test for spatial autocorrelation within sites and among sites. If we changed the site every day, it would have been more difficult to account for spatial autocorrelation.

The experiment was conducted across three winters (Dec. 2016–Mar. 2017, Nov. 2017–Mar. 2017, and Jan. 2018). At each site, we used a different ratio of model-mimics (range: 0–1, 0.2 increments, Table S1). Our daily protocol consisted of deploying prey starting 30-min after sunrise, which took about 60-min to deploy. We left prey for 2 h, after which we collected the prey again and recorded the rates of partial predation (hereafter referred to as ‘taste rejection’). We also filmed a single prey on each day to check which predators were attacking the prey. The camera was set up ~ 4 m from a prey that was chosen randomly each day. When we observed an attack, we calculated the duration of the attack by timing from when the bill first touched the prey until when the bird either finished eating the prey or its bill last touched the prey. Partial predation was when there was an obvious attack and the bird left part of the prey behind. Typically, the bird either punctured the body, removed the head, or ate part of the prey and left the remainder behind. Although we attempted to collect 8 consecutive days of data at each site, we did not run the trials when it was raining. In such instances, we continued the prey deployments on the next day with good weather [see^21^ for more details].

This experiment was approved by the Animal Ethics Experimental Committee of Guangxi University and the Animal Ethics Committee of the Department of Zoology at Kyoto University. The experiment complied with all laws of the countries in which it was conducted and adhered to the Animal Behavior Society/Association for the Study of Animal Behaviour regulations for the use of animals in research. This experiment also adhered to the ARRIVE guidelines.

### Predator diversity survey

Our experiment time was designed to minimize the effects of time of day, time of year, study site, and the effect of biases predators might have had for or against particular colours (Table S1). However, the abundance and diversity of predators might influence the attack rate and taste rejection rate^22,23^. Therefore, before starting the daily trials, we conducted a 5-min bird point-count to determine the assemblage of potential predators^24^, recording the species identity of all birds detected (seen or heard, except those in flight). The bird count was performed at the centre of a 50 m diameter circle of the study site. Bird counts were performed during the 4-day experimental stage [see^21,25^ for further details of bird count methods].

### Statistics

We used generalized linear mixed-effect models (GLMMs) with a binomial distribution to determine how mimic frequency influenced the frequency of taste rejection, by using the *lme4* package^26^. We analysed data from Nanning and Kyoto separately because there was a possibility that the two regions had different results from one another, which might cancel one another out. This also allowed us to better understand the variation in each city. We combined the 4-days taste rejection records and included the proportion of taste rejected prey (taste rejected prey/the total number of prey per sub-site) as the dependent variable. Mimic frequency (0, 0.2, 0.4, 0.6, 0.8, and 1) was the fixed effect, and sub-site nested within study site (6 sites per region) the random factor. We also calculated the level of taste rejection relative to overall predation to account for the effect of predation rate (i.e., proportion of taste rejected prey/proportion of attacked prey; hereafter, ‘relative taste rejection’). We performed a GLMM on this ratio as dependent variable and following the same procedure described above. We then repeated this procedure using data from the controls. We found the models of Nanning’s treatment were completely separated because there was no taste rejection at the mimic frequency of 1.0 in Nanning^27^. Therefore, we added an artificial 1 taste rejection observation to the Nanning data. We also increased the number of iterations to the maximum (10,000) to improve model convergence. We calculated the coefficient of determination statistics to examine the amount of variation explained by the model^28^. Type III Wald chi-square tests were conducted to estimate the significance levels of our independent variables. We conducted post-hoc tests by comparing the pairwise least square means with the P-value adjusted by the Bonferroni method to examine the differences in proportion of (relative) taste rejection among mimic frequencies using the *multcomp* package^29^. Both GLMM models above did not show any evidence of overdispersion and spatial autocorrelation, which was assessed by using Moran I Tests on the model residuals using the “Moran.I” command in the *ape* package^30^. This indicates that there was enough mixing of individual birds within the parks to outweigh any effect of bird territoriality. Finally, we examined attack duration by running another GLMM. Due to the small sample size we could not test the effect of many factors, so we included attack duration as the dependent variable, mimic frequency as the fixed factor, and predator species and city as random variables. We also used Wilcoxon rank sum tests to examine the difference in proportion of taste rejection and proportion of relative taste rejection between the two study regions and between the treatment and the control, respectively.

Since predator diversity could influence attack rate^21–23,31,32^, we tested the dissimilarity of bird composition between study sites using the Jaccard’s and Bray–Curtis’s indexes^33^, calculated in package *vegan*^34^ (see electronic supplementary materials for details). Independent t-tests were performed to examine the difference in point count abundance (log-transformed) and richness (log-transformed) between the two study regions. All analyses were run using R software version 3.4.2^35^.

## Results

### Effect of mimic frequency on taste rejection

**Figure 2:**
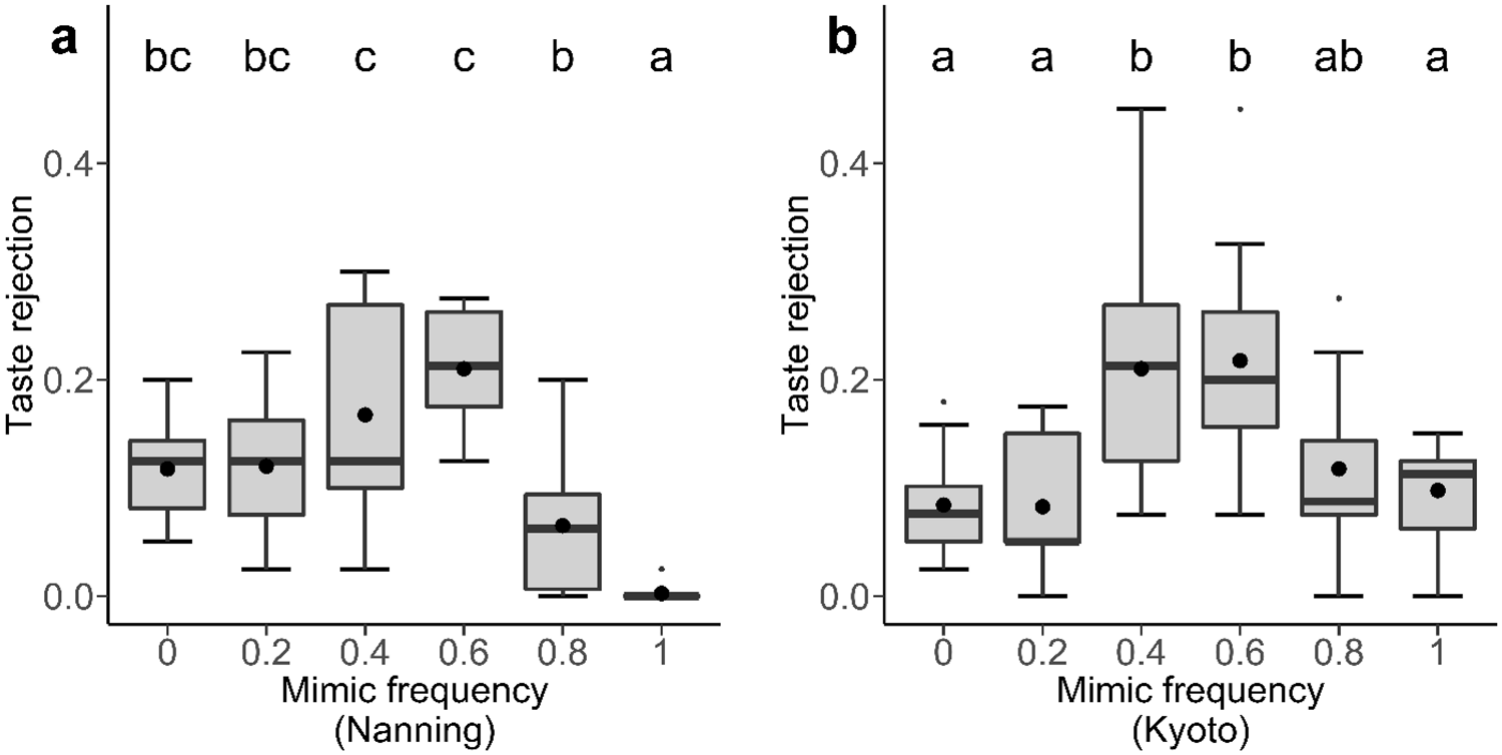
The proportion of prey that were taste rejected by birds at different mimic frequencies in Nanning (**a**) and Kyoto (**b**). The letters indicate commonalities among groups. Black dots represent mean values of each column. Each box represents 10 data points.

**Figure 3:**
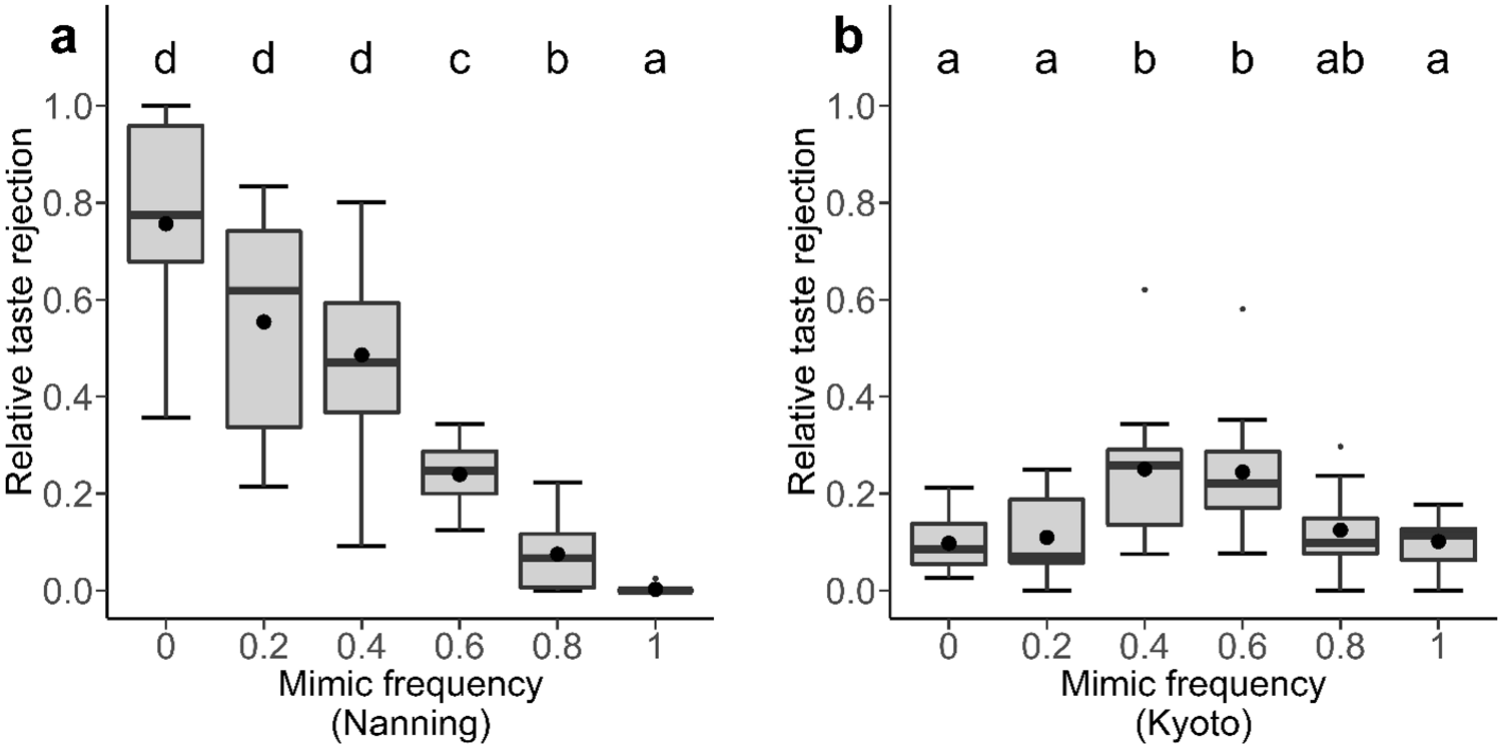
The proportion of attacked prey that were taste rejected by birds at different mimic frequencies in Nanning (**a**) and in Kyoto (**b**). The letters indicate commonalities among groups. Black dots represent mean values of each column. Each box represents 10 data points.

There was a significant effect of mimic frequency on the proportion of prey that were taste rejected in both study regions (Nanning: ${\chi }_{5}^{2}$ = 43.62 *P* < 0.001; Kyoto: ${\chi }_{5}^{2}$ = 30.28, *P* < 0.001, Fig. 2). Crucially, in both study regions the pattern of taste rejection matched our prediction that taste rejection would be greatest when the reliability of the colour signal was lowest (i.e., proportions of prey taste rejected in mimic frequencies 0.4 and 0.6 were significantly higher than in other frequencies, Fig. 2 Table S2). When we examined the amount of relative taste rejection (i.e., taste rejection in relation to total predation), we found a significant effect of mimic frequency (in Nanning: ${\chi }_{5}^{2}$ = 114.26, *P* < 0.001; in Kyoto: ${\chi }_{5}^{2}$ = 25.3, *P* < 0.001). However, only the result from Kyoto matched our predicted pattern (Fig. 3b, Table S2), whereas the result from Nanning showed a downward pattern, in which relative taste rejection was highest when there were no undefended mimics presented (i.e., mimic frequency 0), and lowest when only undefended mimics were presented (i.e., mimic frequency 1) (Fig. 3a, Table S2). We also found that there was a significant effect of mimic frequency on the durations of the attacks (${\chi }_{1}^{2}$ = 4.581, *P* = 0.032, Fig. S3). In Nanning, the results showed that there were no significant effects of mimic frequency on both taste rejection and relative taste rejection of the control results (taste rejection: ${\chi }_{5}^{2}$ = 4.89, *P* = 0.43; relative taste rejection: ${\chi }_{5}^{2}$ = 7.22, *P* = 0.21), whereas results from Kyoto were significant (taste rejection: ${\chi }_{5}^{2}$ = 39.65, *P* < 0.001; relative taste rejection: ${\chi }_{5}^{2}$ = 38.07, *P* < 0.001). However, these analyses revealed that taste rejection was not simply related to the level of predation (Fig. S2, Fig. S4, Table S2).

There was no significant difference in the levels of taste rejection between Nanning and Kyoto (Nanning: mean ± SD 0.114 ± 0.091; Kyoto: mean ± SD, 0.14 ± 0.098; W = 2007.5, *P* = 0.28; Fig. S5a). However, the proportions of relative taste rejection in Nanning were significantly higher than in Kyoto (in Nanning: mean ± SD, 0.35 ± 0.31; in Kyoto: mean ± SD, 0.16 ± 0.12; W = 1231.5, *P* < 0.001; Fig. S5b). In both study regions, the proportion of taste rejection and relative taste rejection of the treatment were higher than their control (taste rejection: in Nanning, mean ± SD, 0.11 ± 0.091 vs. 0.011 ± 0.021; W = 3069, *P* < 0.001; in Kyoto, mean ± SD, 0.14 ± 0.098 vs. 0.055 ± 0.075; W = 2839, *P* < 0.001; relative taste rejection: in Nanning, mean ± SD, 0.35 ± 0.31 vs. 0.015 ± 0.028; W = 3102.5, *P* < 0.001; in Kyoto, mean ± SD, 0.35 ± 0.31 vs. 0.015 ± 0.028; W = 2862.5, *P* < 0.001).

### Differences in avian communities between cities and among sites

The bird communities in Nanning and Kyoto were distinct (Table S3). There were significant differences in bird richness and abundance between our study sites in Nanning and Kyoto, with both metrics being higher in Nanning (log-richness: t = − 6.98, *P* < 0.001; log-abundance: t = − 11.56, *P* < 0.001; Fig. S6). Comparisons of the levels of dissimilarity among sites showed that sites within cities were comparatively less dissimilar from one another compared with sites between cities (Table S3). We recorded 29 species of birds in Nanning, of which only 4 species were observed attacking model-mimics: long-tailed shrikes (*Lanius schach*), oriental magpie-robins (*Copsychus saularis*), red-whiskered bulbuls (*Pycnonotus jocosus*), and sooty-headed bulbuls (*Pycnonotus aurigaster*) (Fig. S1). In Kyoto, we counted 26 species and prey were attacked mostly by Japanese tits (*Parus minor*) and Japanese bush warblers (*Horornis diphone*), although brown-eared bulbuls (*Hypsipetes amaurotis*) also attacked prey occasionally.

## Discussion

In this study, we found that taste sampling varied across the range of model-mimic frequencies in relation to the reliability of the prey’s colour signals at signalling whether a prey was defended or not^2,3^. Taste rejection was greatest at moderate mimic frequencies (0.4 and 0.6) and lowest at low and high mimic frequencies. This confirms our hypothesis that predators taste rejected more prey when colour signals were more unreliable for predators attacking mimetic prey. Moreover, the data for relative taste rejection show that taste rejection was not related to the overall level of predation, meaning that the taste rejection was related to the colour signal and the uncertainty that it created in predators rather than predation levels. This is the first time that this quantitative prediction of the ‘go-slow’ hypothesis has been confirmed and we encourage further examination of how taste rejection relates to mimic frequency, and whether this result is consistent across environments.

Earlier studies have examined predation at different mimic frequencies and have shown that birds exhibit go-slow behaviour^7–9^. For example, it was found that taste rejection of attacked prey was higher when the mimic frequency was 0.5 compared with 0.25^7^. This is consistent with our finding that a higher proportion of the sample prey were rejected when the colour signals of the prey were less accurate in providing information about the prey’s defences. However, our study is the first to find this difference in taste rejection over the entire spectrum of mimic frequencies. Previous authors have argued that the concept of aposematism needs refinement to account for the complexities of avian decision making in relation to the evolution of honest visual signals^36^. Our finding that predators may be able to dynamically change their taste sampling of prey in relation to the level of signal unreliability aids this refinement.

There were low levels of taste rejection of the undefended prey across mimic frequencies. This suggests that birds quickly learned about the prey’s properties and began responding to the model-mimics in an educated manner. Moreover, the birds were not treating the two prey types similarly to one another, nor generalizing the properties of the model-mimics to all prey. Rather, predators learned the properties of the prey and responded to them in appropriate ways by seeking further information through tasting when the visual signals were unreliable^18^. Surprisingly, there was more taste rejection of undefended control prey in Kyoto than in Nanning. The reason for this could have been due to birds attacking the prey and then being disturbed by competitors or predators during the attack. Our data suggest that the predators quickly learned to discount the value of the prey’s visual signals when they were unreliable.

Importantly, we also found that the levels of taste rejection were unrelated to predation rates at different mimic frequencies and levels of defence. If taste rejection was only a reflection of the average level of defence in the model-mimics, we would have expected a high level of relative taste rejection at sites where models were more common and for the level of taste rejection to decline as the mimic densities increased. This is because birds are known to taste reject aposematic prey^18^. However, we found relatively lower levels of taste rejection at low mimic frequencies and high mimic frequencies. At these frequencies, predators either fully consumed prey at high mimic frequencies or eschewed attacking prey at low mimic frequencies.

While we found that taste sampling increased when the visual signals of prey were less reliable, we did not examine the differences for models and mimics separately. Previous studies^7–9^ have found that mimics were eaten at higher rates after predators attacked prey compared to models, which confirms that predators likely taste sample prey to confirm the defensive properties of the prey^17^. Therefore, it is reasonable to expect that in this study, more mimics were eaten than models after they were attacked, because taste sampling allows predators to discriminate between prey with ambiguous warning signals using taste. We also did not estimate prey survival, but previous studies have shown that taste sampled prey can often survive predator attacks^37–40^ and defended prey may be attacked with less force than undefended prey^21,41,42^. Moreover, aposematic species often evolve tough and flexible bodies^43–45^ meaning that models may have higher survival than mimics^3^. This means that although taste sampling is costly for models (e.g., through being injured^39^), it likely exerts a higher cost on mimics, which may reinforce the honesty of the model’s defensive signal^46^.

If the level of taste rejection is related to the overall level of predation experienced by prey at each mimic frequency, we might have expected similar levels of relative taste rejection among the prey at the various mimic frequencies. However, we found that the relative level of taste rejection had different patterns in different regions. In Nanning, the level of relative taste rejection declined as the mimic frequency increased, as is expected by the go-slow hypothesis, in which birds taste and consume mimics, and taste and reject models, making the percentage of rejected prey directly related to the percentage of models in the population. In Kyoto, we found that the pattern of relative taste rejection mirrored the pattern of taste rejection, likely because the days in Kyoto were colder and shorter in the winter time than in Nanning. This likely means the birds in Kyoto were more energetically stressed than birds in Nanning, which would result in higher overall predation^19,47^. Almost all prey were attacked in Kyoto, even when the model-mimics consisted mostly of models. At low mimic frequencies the prey had high signal reliability, leading to low levels of taste rejection. Meanwhile, different energetic needs of the birds in the two cities led to different decisions (i.e., reject the prey in Nanning and accept them in Kyoto). This caused similar levels of taste rejection (taste rejection/total prey), but different levels of relative taste rejection (taste rejection/predation) between the two study regions. This is an extreme situation which illustrates how predators may change their behaviour in response to environmental differences. However, classical Batesian/auto mimicry and ‘go-slow’ theories did not consider how foraging preferences might change according to environmental variance.

Interestingly, we also found that our result was unaffected by the bird communities at each of the sites despite significant differences in the communities between regions. This suggests that, irrespective of the predator community, predatory birds behave in the same way when faced with this constraint of information. Other studies have demonstrated the importance of the avian community as a selective force on aposematic prey^22,23^. However, we were not recording predation of single species, rather we recorded predation of a community of individuals. It is likely that there are differences in tolerance to toxins among individuals of different species. For example, it was recently shown that Japanese tits (*Parus minor*) showed no preference for undefended prey over chemically defended prey, whereas two other bird species had a strong preference for undefended prey^31^. It was likely this difference in preference among species was due to Japanese tits having higher tolerances for the defensive compounds used in this experiment. Therefore, the composition of the predator community is likely to influence predation of aposematic prey and their mimics.

Overall, we have shown that birds taste reject prey in relation to the levels of uncertainty that are associated with the prey’s visual signal. Predators taste sampled prey when the reliability of prey colour signal is low and eschewed taste sampling when signal reliability is high. However, the relationships between the reliability of visual signals, prey toxicity and their taste to predators are complex^18,47,48^. Although it had been shown previously that predators display ‘go-slow’ behaviour^7–9^, no study had assessed whether taste sampling is related to the frequency of mimics and the levels of signal reliability of the prey across the entire mimicry spectrum and in different climatic zones. This indicates that birds respond to unreliable prey signals similarly across different climatic zones and environments. Moreover, taste sampling offers a way for predators to circumvent unreliable signals and increase their ability to reject defended models when colour signals are most unreliable.

## Supplementary Information

Supplementary Information.
