# Supplementary material for: Avian predators taste reject mimetic prey in relation to their signal reliability
Source: Sci Rep. 2022 Feb 11;12:2334. doi: 10.1038/s41598-022-05600-5 (PMC8837650; doi:10.1038/s41598-022-05600-5)
Supplement: Supplementary file 1 — Supplementary Information. [file 41598_2022_5600_MOESM1_ESM.docx]

**Supplementary information for He et al. “Avian predators taste reject mimetic prey in relation to their signal reliability”**

**Table S1.** The locations of the study sites, mimic frequencies, and the prey colours. The order of the experiments, and the assignment of a mimic frequency to a park, was randomised, and the colours were assigned systematically to balance the design of the experiment.

|  |  |  | Colour | |  |
| --- | --- | --- | --- | --- | --- |
| Date | Locations | Study site | Control | Treatment | Mimic frequency |
| Dec, 2016 - Jan, 2017 | 22°86’ N, 108°34’ E | Shishan Park (SP) | Pink | Yellow | 0 |
| Jan - Feb, 2017 | 22°84’ N, 108°28’ E | Xinxu river Park (XRP) | Pink | Yellow | 0.8 |
| Feb, 2017 | 22°83’ N, 108°32’ E | Peoples’ Park (PP) | Pink | Yellow | 0.4 |
| Mar, 2017 | 22°86’ N, 108°37’ E | Medicinal Botanical Garden (MBG) | Yellow | Pink | 0.2 |
| Nov, 2017 | 22°86’ N, 108°29’ E | Flower Park (FP) | Yellow | Pink | 1 |
| Dec, 2017 | 22°84’ N, 108°24’ E | Xiangsi Lake Park (XLP) | Yellow | Pink | 0.6 |
| Nov, 2016 | 35°01’ N, 135°48’ E | Yoshidayama (YM) | Pink | Yellow | 0.2 |
| Dec, 2016 | 35°02’ N, 135°49’ E | Fudo Onsen (FO) | Yellow | Pink | 0.8 |
| Dec, 2016 | 35°03’ N, 135°49’ E | Manshu-in (MI) | Yellow | Pink | 0.4 |
| Jan, 2017 | 35°03’ N, 135°47’ E | Takara-ga-ike 1 (TGI1) | Yellow | Pink | 0 |
| Dec, 2017 | 35°03’ N, 135°46’ E | Takara-ga-ike 2 (TGI2) | Pink | Yellow | 0.6 |
| Jan, 2018 | 35°02’ N, 135°46’ E | Shimagamo Jinga (SJ) | Pink | Yellow | 1 |

**Table S2.** P-values for post-hoc comparisons among the differences in taste rejection among the different mimic frequencies.

|  |  | **Taste rejection** | | | **Relative taste rejection** | |
| --- | --- | --- | --- | --- | --- | --- |
| **Nanning** |  | Treatment  R^2^m=0.43 R^2^c=0.45 | Control (n.s.)  R^2^m=0.11 R^2^c=0.36 | Treatment  R^2^m=0.61 R^2^c=0.65 | | Control (n.s.)  R^2^m=0.14 R^2^c=0.4 |
|  | 0 vs 0.2 | 1 | **-** | 0.34 | | **-** |
|  | 0 vs 0.4 | 0.6 | - | 0.1 | | - |
|  | 0 vs 0.6 | 0.051 | **-** | **< 0.001** | | **-** |
|  | 0 vs 0.8 | 0.2 | - | **< 0.001** | | - |
|  | 0 vs 1 | **0.0012** | **-** | **< 0.001** | | **-** |
|  | 0.2 vs 0.4 | 0.65 | - | 0.99 | | - |
|  | 0.2 vs 0.6 | 0.062 | - | **0.0027** | | - |
|  | 0.2 vs 0.8 | 0.17 | - | **< 0.001** | | - |
|  | 0.2 vs 1 | **0.0011** | **-** | **< 0.001** | | **-** |
|  | 0.4 vs 0.6 | 0.79 | **-** | **0.0095** | | **-** |
|  | 0.4 vs 0.8 | **0.0025** | - | **< 0.001** | | - |
|  | 0.4 vs 1 | **< 0.001** | **-** | **< 0.001** | | **-** |
|  | 0.6 vs 0.8 | **< 0.001** | **-** | **< 0.001** | | **-** |
|  | 0.6 vs 1 | **< 0.001** | - | **< 0.001** | | - |
|  | 0.8 vs 1 | **0.01411** | **-** | **0.013** | | **-** |
| **Kyoto** |  | Treatment  R^2^m=0.3002 R^2^c=0.3369 | Control  R^2^m=0.2524 R^2^c=0.3824 | Treatment  R^2^m=0.065 R^2^c=0.155 | | Control  R^2^m=0.211 R^2^c=0.34 |
|  | 0 vs 0.2 | 1 | 0.72 | 1 | | 0.72 |
|  | 0 vs 0.4 | **0.0069** | 0.33 | **0.017** | | 0.39 |
|  | 0 vs 0.6 | **0.0038** | **0.0045** | **0.019** | | **0.0057** |
|  | 0 vs 0.8 | 0.88 | 0.97 | 0.98 | | 0.98 |
|  | 0 vs 1 | 1 | **< 0.001** | 1 | | **< 0.001** |
|  | 0.2 vs 0.4 | **0.0045** | 0.98 | **0.025** | | 0.99 |
|  | 0.2 vs 0.6 | **0.0025** | 0.084 | **0.027** | | 0.11 |
|  | 0.2 vs 0.8 | 0.82 | 0.99 | 0.99 | | 0.98 |
|  | 0.2 vs 1 | 0.99 | **0.0015** | 1 | | **0.0023** |
|  | 0.4 vs 0.6 | 1 | 0.32 | 1 | | 0.31 |
|  | 0.4 vs 0.8 | 0.15 | 0.77 | 0.1 | | 0.79 |
|  | 0.4 vs 1 | **0.033** | **0.011** | **0.02** | | **0.011** |
|  | 0.6 vs 0.8 | 0.1 | **0.02** | 0.11 | | **0.02** |
|  | 0.6 vs 1 | **0.02** | 0.71 | **0.023** | | 0.73 |
|  | 0.8 vs 1 | 0.99 | **< 0.001** | 0.99 | | **< 0.001** |

**Table S3.** Bray–Curtis's and Jaccard's distance were calculated to examine the dissimilarity of bird diversity among 12 study sites (4 day’s point count data were combined).

|  | | | Nanning | | | | | | Kyoto | | | | |
| --- | --- | --- | --- | --- | --- | --- | --- | --- | --- | --- | --- | --- | --- |
|  |  |  | SP | MBG | PP | XLP | XRP | FP | TGI1 | YM | MI | TGI2 | FO |
| Jaccard's index | Nanning | MBG | 0.49 |  |  |  |  |  |  |  |  |  |  |
|  |  | PP | 0.65 | 0.59 |  |  |  |  |  |  |  |  |  |
|  |  | XLP | 0.60 | 0.60 | 0.62 |  |  |  |  |  |  |  |  |
|  |  | XRP | 0.60 | 0.59 | 0.68 | 0.51 |  |  |  |  |  |  |  |
|  |  | FP | 0.50 | 0.61 | 0.68 | 0.60 | 0.58 |  |  |  |  |  |  |
|  | Kyoto | TGI1 | 0.99 | 0.99 | 0.99 | 0.99 | 1.00 | 0.99 |  |  |  |  |  |
|  |  | YM | 0.99 | 0.99 | 0.99 | 0.99 | 1.00 | 0.99 | 0.35 |  |  |  |  |
|  |  | MI | 0.99 | 0.99 | 0.99 | 0.99 | 1.00 | 0.99 | 0.74 | 0.77 |  |  |  |
|  |  | TGI2 | 0.98 | 0.98 | 0.97 | 0.98 | 1.00 | 0.98 | 0.50 | 0.48 | 0.68 |  |  |
|  |  | FO | 1.00 | 1.00 | 1.00 | 1.00 | 1.00 | 1.00 | 0.64 | 0.62 | 0.76 | 0.80 |  |
|  |  | SJ | 1.00 | 1.00 | 1.00 | 1.00 | 1.00 | 1.00 | 0.66 | 0.59 | 0.80 | 0.62 | 0.76 |
| Bray–Curtis's index | Nanning | MBG | 0.32 |  |  |  |  |  |  |  |  |  |  |
|  |  | PP | 0.48 | 0.42 |  |  |  |  |  |  |  |  |  |
|  |  | XLP | 0.43 | 0.43 | 0.45 |  |  |  |  |  |  |  |  |
|  |  | XRP | 0.43 | 0.42 | 0.52 | 0.34 |  |  |  |  |  |  |  |
|  |  | FP | 0.34 | 0.44 | 0.51 | 0.43 | 0.40 |  |  |  |  |  |  |
|  | Kyoto | TGI1 | 0.98 | 0.98 | 0.98 | 0.98 | 1.00 | 0.98 |  |  |  |  |  |
|  |  | YM | 0.99 | 0.98 | 0.98 | 0.98 | 1.00 | 0.98 | 0.22 |  |  |  |  |
|  |  | MI | 0.99 | 0.98 | 0.98 | 0.98 | 1.00 | 0.98 | 0.58 | 0.62 |  |  |  |
|  |  | TGI2 | 0.97 | 0.96 | 0.95 | 0.95 | 1.00 | 0.97 | 0.33 | 0.32 | 0.51 |  |  |
|  |  | FO | 1.00 | 1.00 | 1.00 | 1.00 | 1.00 | 1.00 | 0.47 | 0.45 | 0.61 | 0.66 |  |
|  |  | SJ | 1.00 | 1.00 | 1.00 | 1.00 | 1.00 | 1.00 | 0.49 | 0.42 | 0.67 | 0.45 | 0.61 |

Supplementary figure legends

**Figure S1.** Photographs depicting a red-whiskered bulbul (*Pycnonotus jocosus*) (**a**) and an oriental magpie-robin (*Copsychus saularis*) (**b**) attacking the prey, and examples of prey that were rejected after tasting the prey (**c** and **d**).

**Figure S2.** The proportion of alternative (control) prey that were taste rejected by birds at different mimic frequencies in Nanning (**a**) and Kyoto (**b**). The letters indicate commonalities among groups. Black dots represent mean of each column. Each box represents 10 data points.

**Figure S3.** The length of time that birds spent attacking prey (s) based on a subsample of prey at which we filmed attacks. Overall, we filmed 34 attacks in total (Kyoto *n* = 13, Nanning *n* = 21) which we combined in our analysis.

**Figure S4.** The relative levels of taste rejection of alternative (control) prey at different mimic frequencies in Nanning (**a**) and Kyoto (**b**). The letters indicate commonalities among groups. Black dots represent mean of each column. Each box represents 10 data points.

**Figure S5.** The proportion of taste rejection (**a**) and relative taste rejection (**b**) in Nanning and Kyoto. Black dots represent mean of each column. Each box represents 60 data points.

**Figure S6.** Bird richness (**a**) and abundance (**b**) in Nanning and Kyoto. Black dots represent mean of each column. Each box represents 4 data points from point-counts.

**Figure S1**


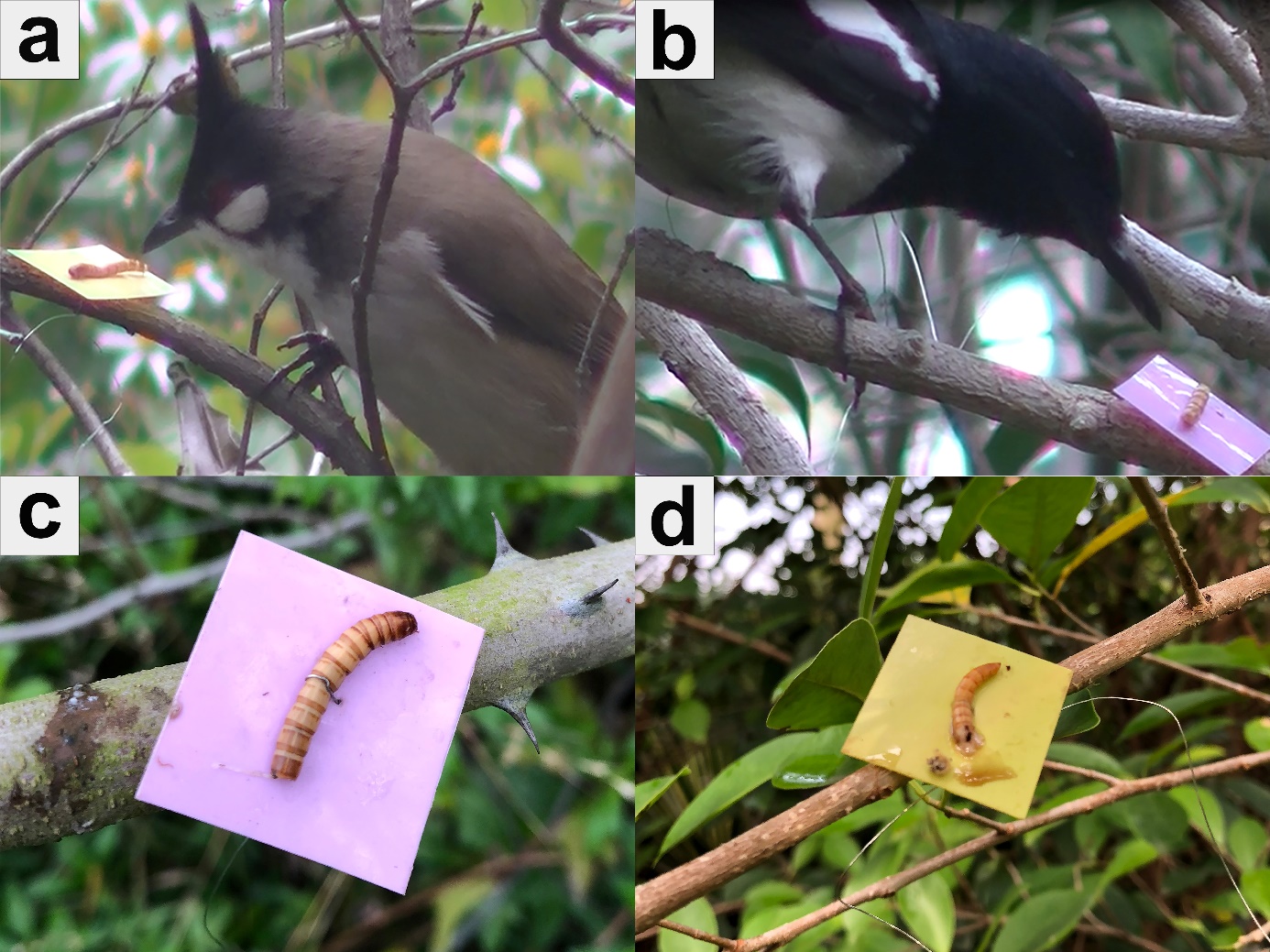


**Figure S2**

**
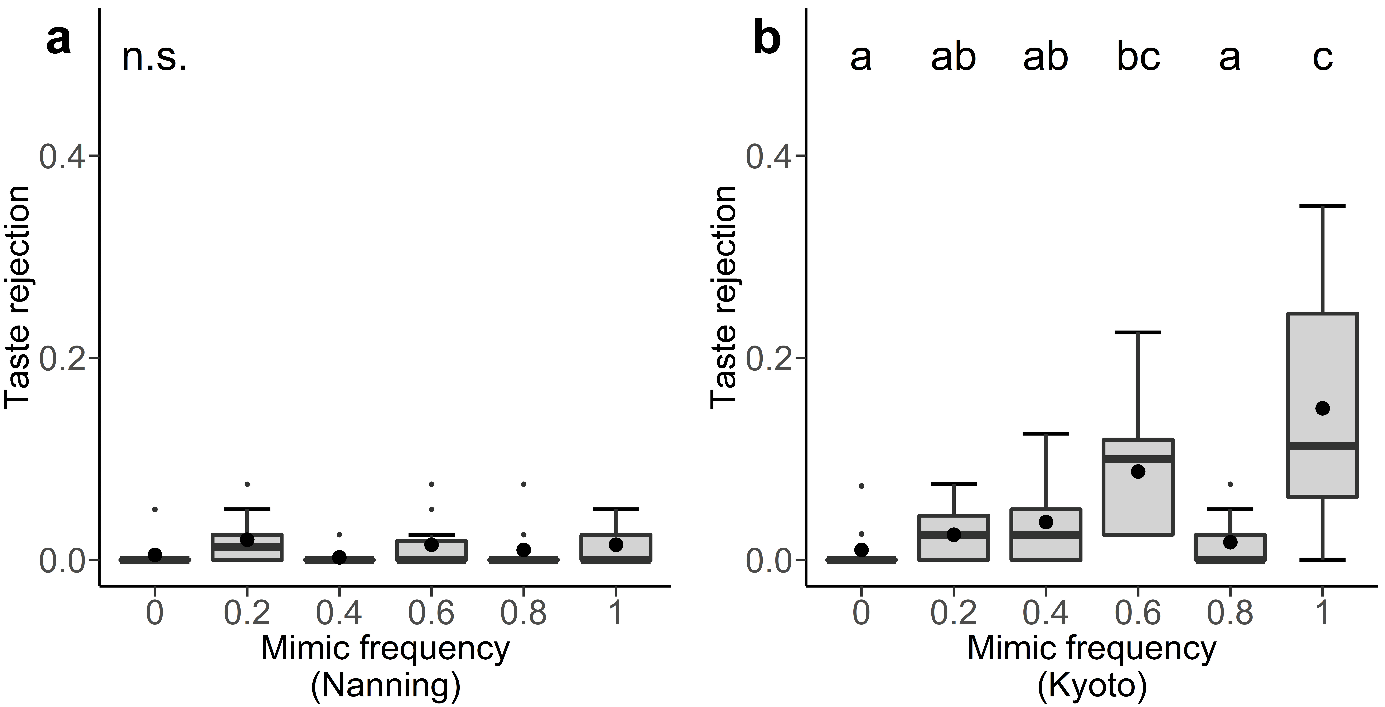
**

**Figure S3**

*
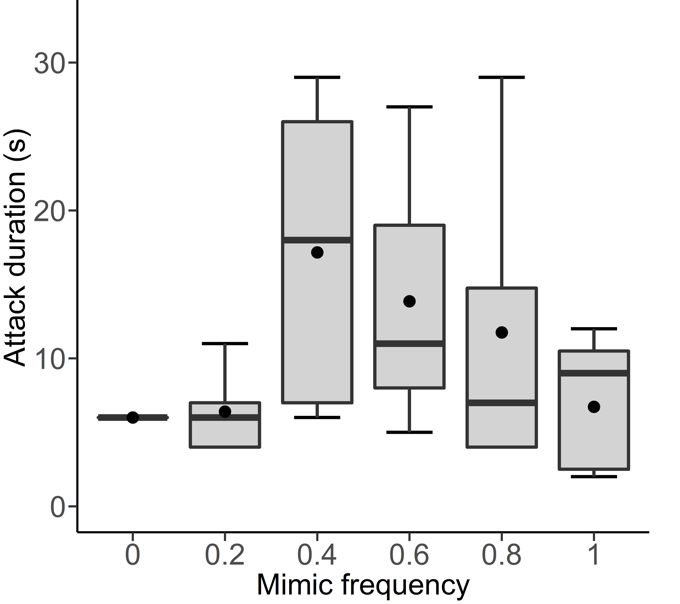
*

**Figure S4**

**
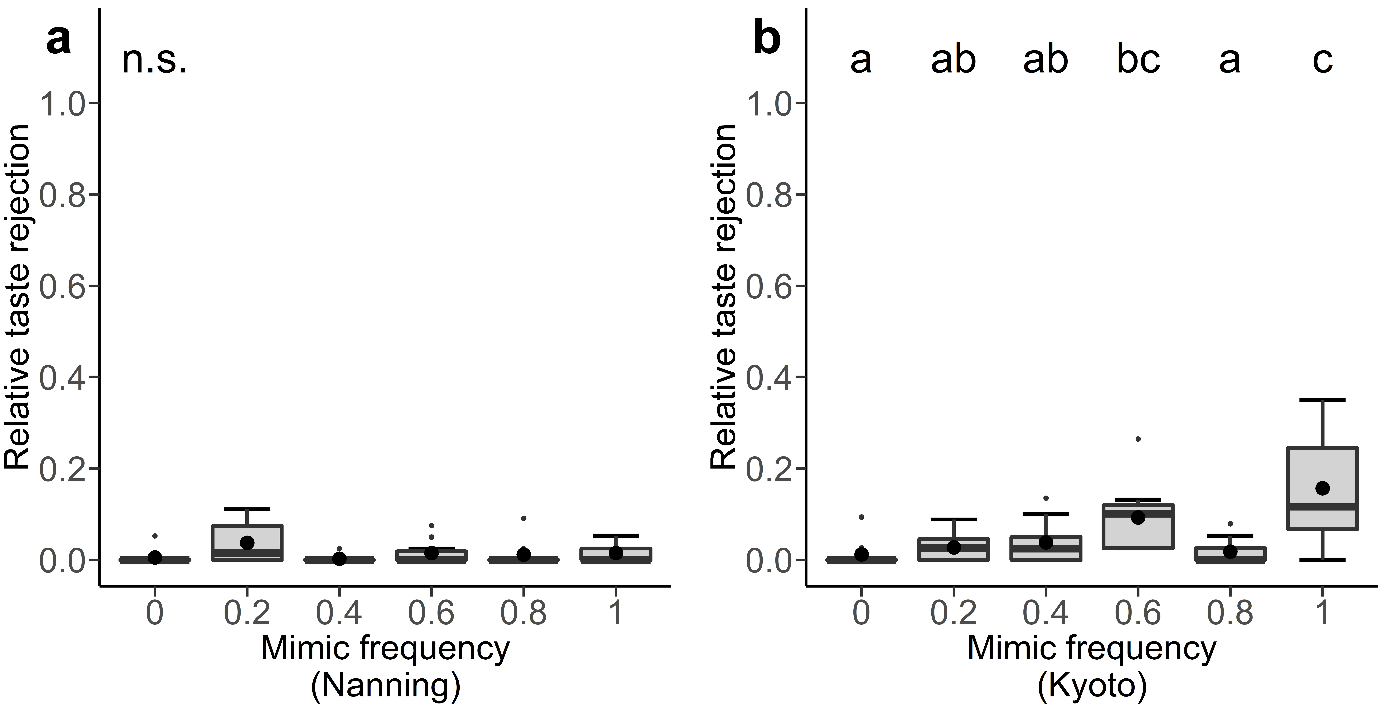
**

**Figure S5**

**
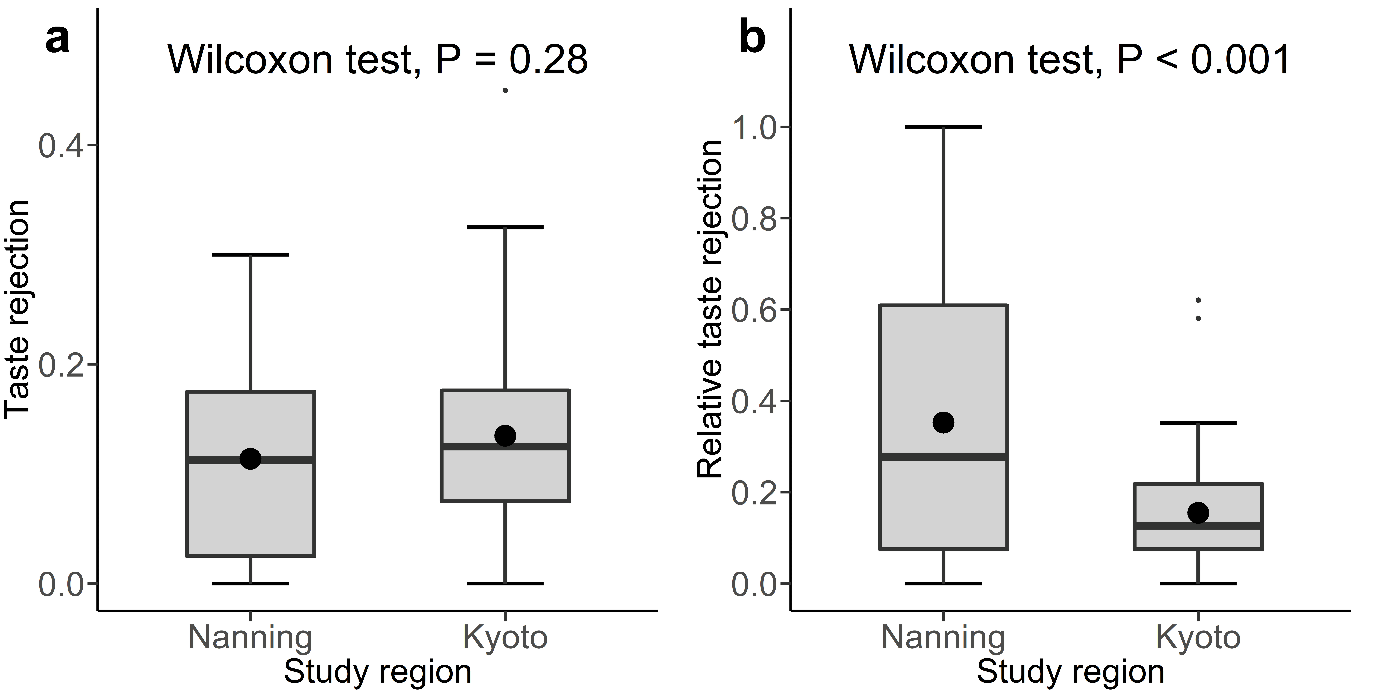
**

**Figure S6**

**
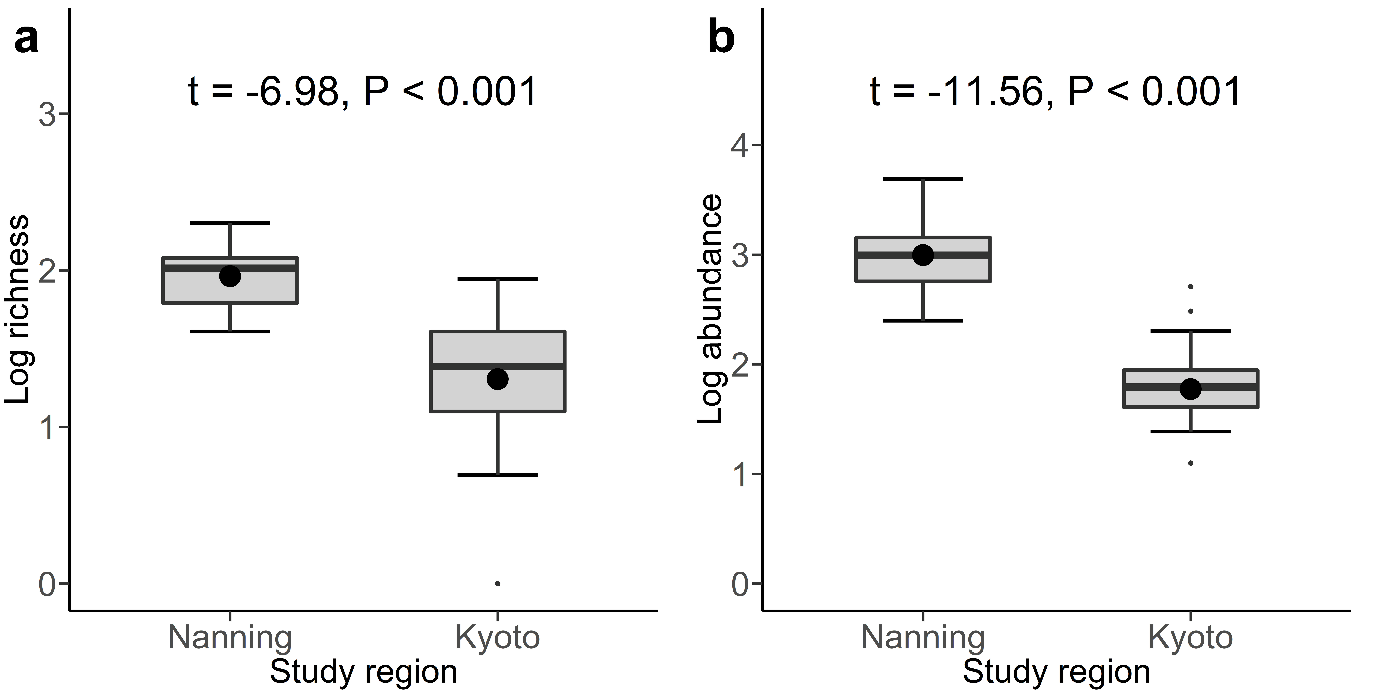
**
